# Efficacy and Safety of Q-Switched 1064/532 nm Nd:YAG Lasers on Benign Hypermelanosis in Dark-Skinned Individuals—A Preliminary Study

**DOI:** 10.3390/jcm13061615

**Published:** 2024-03-12

**Authors:** Domenico Piccolo, Irene Fusco, Giuliana Crisman, Tiziano Zingoni, Claudio Conforti

**Affiliations:** 1 Novea Skin Center-Dermo Aesthetic Laser Centers, 67051 Avezzano, Italy; domenico.piccolo.skincenters@gmail.com (D.P.); giuliana.crisman@gmail.com (G.C.); 2 El.En. Group, 50041 Calenzano, Italy; t.zingoni@elen.it; 3 IDI-IRCCS, Dermatological Research Hospital, 00167 Rome, Italy; claudioconforti@yahoo.com

**Keywords:** benign hypermelanosis, dark-skinned individuals, 1064/532 nm Q-switched laser

## Abstract

**Background**: Facial hypermelanosis is a major cosmetic issue that causes severe social embarrassment and psychological pain, particularly among Asians and dark-skinned individuals. **Aim**: This study assesses the safety and effectiveness of Q-switched 1064/532 nm nanosecond/picosecond lasers in removing benign hypermelanosis in dark-skinned individuals, evaluating the possible associated side effects. **Material and methods**: A total of 30 participants (80% females and 20% males) with Fitzpatrick skin types IV–V–VI who presented superficial benign hypermelanoses on the facial and décolleté area were enrolled. All patients underwent to one to two laser treatment sessions with a 1064/532 nm Q-switched laser system. Three months after the final laser session, results were assessed by comparing before- and after-treatment photos and using a quartile scale for lesion clearance (4-point Investigator Global Assessment scale). **Results**: All patients observed global improvements in their pigmented lesions: 53% of patients achieved excellent clearance, 30% of patients achieved good to moderate clearance, 10% of patients achieved slight clearance, and 7% of patients did not respond to the therapy. No serious adverse event occurred. Photos showed the clinical improvement achieved at 3 months follow-up. **Conclusions**: The Q-switched 1064/532 nm laser proved to be a key tool for treating benign hypermelanosis in all skin types, including dark-skinned persons.

## 1. Introduction

Facial hypermelanosis is a major cosmetic issue that causes severe social embarrassment and psychological pain, particularly among Asians and dark-skinned individuals.

Hyperpigmentation, which happens when a skin region is darker than its surroundings, is typical in people with dark skin tones. In these subjects more than in lighter-toned ones, hyperpigmentation usually lasts longer and is harder to treat. Over 65% of African Americans, according to the Skin of Color Society, experience hyperpigmentation signs because of skin damage or irritation [1]. Mature dark skin can show signs of darkening of the face skin tone, even in the absence of significant sun exposure. The term “maturational dyschromia” refers to a general uneven tone or diffuse hyperpigmentation that usually affects the cheekbones and lateral forehead. According to a questionnaire, over one-third of black women reported that their main complaint was uneven skin tone [2]. These skin tone alterations are most likely due to years of continuous sun exposure. It is possible to misdiagnose maturational dyschromia as post-inflammatory hyperpigmentation (PIH), acanthosis nigricans, or melasma. Since this is an exclusion diagnosis, any manifestation of allergic contact dermatitis or photoallergic dermatitis must be ruled out. For cosmetic experts, treating pigmented lesions is one of the most challenging tasks. Excision of such lesions for cosmetic reasons is a surgery that is becoming more common in dermatological procedures. Hyperpigmentation is defined as skin darkening brought on by a melanin deposit in the epidermis or dermis [3]. Pigment production is a complex process that is involved in inflammation, UV protection, and a variety of other processes. Numerous investigations have demonstrated that skin pigmentation alterations can be induced by light from both the UV and visible spectrums [4,5]. Considering clinical experience and existing research, broad-spectrum UVB protection with an SPF of at least 30, ideally incorporating a physical block (such as zinc oxide or titanium dioxide), and UVA-protective sunscreens should be used to avoid facial hyperpigmentation. It is important to properly educate patients when promoting meticulous sun protection, since skin synthesis is a recognized source of vitamin D. Vitamin D is also vital for bone health. Individuals with darker skin naturally have lower vitamin D levels, and those who live in places with little to no sun exposure or who wear strict sun protection measures would need to be closely monitored and supplemented.

Hyperpigmentation is most common in Fitzpatrick skin types III to VI, and it can have a substantial negative impact on quality of life. Particularly, the treatment of pigmented lesions in dark-skinned individuals can be difficult and distressing psychologically. Hyperpigmentations are frequently identified and managed in medical settings.

Freckles, melasma, and post-inflammatory hyperpigmentation are the most prevalent pigmentation disorders. Although these lesions are usually benign, patients may occasionally experience distress. It is possible to rule out malignant tumors and their aftereffects with the use of a comprehensive dermatological history and skin examination.

In actuality, a proper physical and dermatologic history, skin examination, and skin biopsy are required for the diagnosis of hyperpigmentation conditions. To observe significant clinical parameters such as the degree of pigmentary abnormality, distribution, pattern, color hue, and morphology of individual lesions, a thorough skin examination should be carried out in the presence of visible light. When exposed to natural light, dermal hypermelanosis has a less defined edge and a bluish or ashen grey hue, whereas epidermal hypermelanosis appears light brown to dark brown [6].

The period of onset should be discussed in the patient’s dermatologic history since certain medical conditions are congenital, while others (like melasma) arise in childhood, during pregnancy, or in the middle of middle age. Any triggering factor, such as the use of oral contraceptives before melasma onset, inflammatory symptoms, signs before PIH, or the use of topical or oral supplements in previous years, can potentially offer information regarding the diagnosis. Certain traits common to several hyperpigmentary illnesses can be identified by examination. The presence of blue or grey color instead of brown would indicate that the pigmentation is in the dermis, which is supported by Wood’s lamp test, which shows less enhanced pigmentation in the dermal pigmentation and more enhanced pigmentation in the epidermal pigmentation [7].

The lack of a universally effective medication and the varying degrees of effectiveness of existing treatments make managing hyperpigmentation complicated. It is difficult to evaluate the effectiveness of different therapeutic modalities, as most treatment reports are made up of anecdotes and small series of patients. Furthermore, while there are numerous alternatives available today, some medicines have come under greater criticism, emphasizing the relevance of research into pathophysiology and treatment. In general, topical medications or physical modalities are used in conjunction with photoprotection, types of active pigment reduction, and elimination of aggravating variables to provide treatment [8]. Several approaches, including chemical ablation, surgery, and clarifying creams, have been suggested for the management of these melanoses [9]. Moreover, topical therapies for conditions causing facial hyperpigmentation try to interfere with the melanocytes’ enzymatic processes that produce pigment. During the production of melanin, l-tyrosinase is converted to l-3,4-dihydroxyphenlalanine (l-DOPA) by the rate-limiting enzyme tyrosinase. A necessary cofactor for tyrosinase is l-DOPA, and another significant chemical that interacts with the enzyme’s active site is copper. Numerous substances target these molecules, reducing the amount of melanization [10]. Among these, bleaching agents with phenolic compounds such as hydroquinone are often used. However, the negative aspects of hydroquinone-containing bleaching formulas include a high recurrence rate, side effects such as contact dermatitis irritation or a danger of exogenous ochronosis, and the need for a high dosage, and it is not effective in all cases [11]. Adverse outcomes may occur more frequently if high amounts of hydroquinone are accessed uncontrollably or if it is used excessively. Furthermore, the use of topical hydroquinone has raised concerns over the last few years, such as the potential risks associated with benzene derivatives during hepatic metabolism. These compounds are believed to have antiapoptotic and bone marrow toxicity-inducing properties [12]. 

Retinoids have been demonstrated to be advantageous when mixed with other medications, including hydroquinone, lactic acid, and ascorbic acid. However, irritation is the most common adverse effect and can even result in hyperpigmentation, requiring caution when using them [8]. Chemical peels are another type of physical therapy that can be performed alone or in conjunction with other modalities to treat facial hyperpigmentation. Chemical peels can help conditions associated with hyperpigmentation, but they can also irritate skin, which can worsen dyspigmentation. Furthermore, since there is a significant chance of long-lasting and/or permanent pigmentary modifications through peels, they are not usually suggested [13]. Pigmented lesions can be treated using a variety of laser types. The majority of lasers used for these purposes operate on the selective photothermolysis concept [14]. The most effective and secure treatment for benign hyperpigmentation is provided by these lasers, which selectively destroy melanosomes via photomechanical and low-heat effects while protecting adjacent tissues [15]. While the majority of current research supports using this sort of laser on Caucasian populations, a growing amount of scientific data indicate that persons of Asian ethnicity, particularly those with lighter phototypes, may benefit from it as well [15]. Pigmented lesions can be removed with a wide variety of laser and IPL (Intense Pulsed Light) equipment. For this purpose, we can categorize lasers into millisecond, nanosecond, and picosecond technologies. Er:Glass, Er:YAG continuous wave, CO_2_ fractional lasers, dye lasers, and fractional lasers are some of the millisecond technology-based hyperpigmentation therapies available. Nd:YAG and alexandrite lasers are examples of lasers that use nanosecond technology (also known as “Qswitch”), whereas Nd:YAG and alexandrite lasers are the newest advancements in picosecond technology. Every one of these laser kinds has distinct qualities of its own that influence the treatment’s outcome, security, and recovery period [16]. Sub-ablative lasers like the Er:Glass non-ablative fractional lasers, as well as relative fractional lasers like the CO_2_ and Er:YAG lasers, function by creating microchannels in the skin that enable extensive skin remodeling and lightening of discolored areas. Fractional lasers avoid problems and shorten recovery times by carefully targeting specific areas of skin [17]. Before recommending a therapeutic approach, and laser treatment specifically, it is crucial to consider the location of these pigments within the skin, their types that correspond to separated chromophores, and the pathophysiological processes underlying these pigmentary disorders [18]. Indeed, the chromophore and location of a particular skin lesion are two parameters that affect which type of laser is most suitable [19]. As a result, optimal wavelengths with the least amount of hemoglobin or water absorption will target the pigment (typically melanin). The laser pulse duration must be ten times shorter than the target’s thermal relaxation time to provide a selective effect. The vast majority of hyperpigmented diseases are directed against melanosomes, which contain melanin. Since they are just 0.5 µm in size, their relaxation times vary from 1 to 10 µs. Less than 100 nanoseconds should be the ideal pulse duration. Since Q-switched (QS) lasers provide such short pulse durations, they are typically employed for hyperpigmented lesions. Q-switched Nd:YAG lasers (QSNY) at 1064 nm and 532 nm, Q-switched ruby laser (QSRL) at 694 nm and Q-switched alexandrite laser (QSAL) at 755 nm are the primary pigmented lasers in use nowadays [8]. QS lasers are the most efficient and secure method of treating cutaneous hypermelanocytosis due to their exceptional capacity to generate ultra-short (nanosecond) range pulses with high peak power [20]. These lasers act only on the melanin chromophore, sparing adjacent tissues, and deliver high energy in the nano- or picosecond range [18,21,22,23]. As reported in the study of Hałasiński et colleagues [24], there are advantages and disadvantages between nanosecond and picosecond lasers in the treatment of skin and subcutaneous lesions. Specifically, nanosecond lasers selectively target melanin lesions [25], while the use of picosecond lasers achieved good results in removing acne scarring and had a lower rate of adverse reactions for tattoo pigment removal [26]. As for disadvantages, nanosecond lasers produce inferior results in melasma and non-pigmented changes [27], while picosecond lasers have higher treatment costs [28].

Particularly, the Q-switched 1064/532 nm Nd:YAG laser has proved an effective and safe technique for the treatment of pigmented skin lesions as already demonstrated in the literature [15,29,30]. 

Q-switched laser systems, which have a wavelength of 532–1064 nm and a pulse length of picoseconds or nanoseconds, develop a large quantity of energy rapidly [3].

Several published studies have demonstrated the efficacy of Q-switched Nd:YAG lasers in the treatment of different types of pigmented lesions. Some authors [31,32,33] reported the successful treatment of pigmented labial macules seen in Peutz–Jeghers syndrome using a Q-switched Nd:YAG laser. The lesion quickly and completely resolved without causing any textural alterations. A three-center trial [34] showed that a Q-switched neodymium Nd:YAG laser, using the nanosecond 532 nm wavelength with a spot size of 2.0 mm, safely and effectively treated benign epidermal pigmented lesions with a single treatment. In the study of Suh et al. [35], it was observed that a total of 10 (83%) out of 12 patients with freckles showed good to excellent responses to treatment with the Q-switched Nd:YAG laser. 

Additionally, a case report on the use of QS lasers to treat Nevus of Ota was recently published [30]. It demonstrated the effectiveness of this treatment, probably requiring fewer sessions than usual, at lessening the harmful effects of recurrent laser stimulation on the skin. In fact, with just two Q-switched laser sessions performed a year apart, the outcome was excellent, showing a 95% clinical response without adverse effects. However, few studies are available on the effectiveness of Q-switched lasers in removing benign hypermelanosis in dark-skin phototypes. Indeed, recent studies have been very promising in the treatment of hyperpigmentation and melasma in Asian skin but there has been limited research on darker skin types [36,37]. Further information is required on skin color before recommendations can be made.

Based on these findings, this study looks at the effectiveness and safety of Q-switched 1064/532 nm nanosecond/picosecond lasers in removing benign hypermelanosis in dark-skinned individuals, evaluating the possible associated side effects.

## 2. Materials and Methods

This study included 30 participants (80% females and 20% males), ranging in age from 18 to 61 years old and with Fitzpatrick skin types IV–V–VI. The patients presented superficial benign hypermelanoses on the facial and décolleté area which were diagnosed with the use of dermatoscopic evaluation. (see Table 1). Exclusion criteria included hypersensitivity to light (visible and near-infrared); gold-containing medication; therapies with anticoagulants and/or immunosuppressants; medication known to increase sensitivity to light; pregnancy or nursing; sun exposure in the three weeks before treatment (for any skin type); personal or family history of skin cancer; previous hyperpigmentation removal treatment; current skin care procedures (exfoliation); surgical procedures; and previous skin disorders (including keloids). 

A 1064/532 nm Q-switched laser system (SmartPico, Deka M.E.L.A., Calenzano, Italy) was used to treat the patients. It provides Nano/Pico pulses that selectively photothermolyze melanin while causing minimal thermal damage to surrounding biological tissues. The system can be equipped with a skin contact sensor.

A skin test was conducted before the beginning of the therapy to determine the appropriate treatment parameter values based on the skin type of the subject. All patients underwent to 1–2 laser treatment sessions. External air cooling before and after treatment was performed. The treatment parameters were as follows: for lighter lesions or which are in the process of clearing, picosecond 532 nm, fluence 0.4–0.8 J/cm^2^, frequency 1–2 Hz, and spot size 3–6 mm. For darker lesions, the parameters were nanosecond 532 nm, fluence 0.8–1.4 J/cm^2^, frequency 1–2 Hz, and spot size 3–6 mm.

Laser treatments were conducted at least 30 days apart or until the skin fully recovered. Three months after the last laser treatment session, the final evaluation and follow-up visits occurred; the total removal of the benign hyperpigmented lesion(s) was the clinical endpoint for the therapy.

Each patient signed an informed consent form agreeing with the procedure’s risks. Three months following the last laser session, a dermatologist evaluated the results by comparing pre- and post-treatment photos. The device’s performance was assessed by classifying the results into four categories based on a quartile scale of lesion clearance (4-point Investigator Global Assessment scale): 1 = no or low results (0–25% of the lesion area improved), 2 = slight improvement (25–50% of the lesion area cleared), 3 = moderate to good improvement (50–75%), and 4 = excellent improvement (75–100%). Before the first treatment and three months after the last session, digital and three-dimensional (3D) photographs were obtained from each patient by using two different 3D digital cameras (Vectra H2, Canfield, OH, USA, and LifeViz Mini, Quantificare, Biot, France), to evaluate the aesthetic improvement of the patient’s pigmented lesions.

As postoperative care for the patients, the use of a broad-spectrum sunscreen was recommended.

## 3. Results

**Figure 1:**
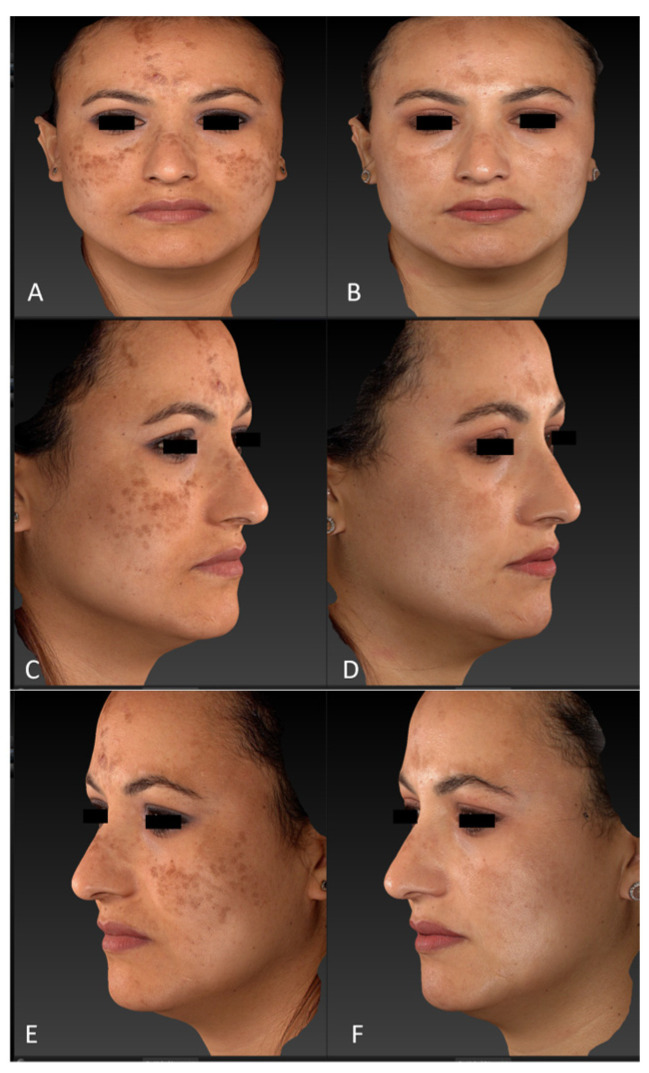
Diffuse hypermelanoses on a female patient’s face area (cheeks, forehead, and nose) before treatment (frontal view, (**A**); right lateral view, (**C**); left lateral view, (**E**)) and three months after the last laser treatment session (frontal view, (**B**); right lateral view, (**D**); left lateral view, (**F**)). Images acquired with a Vectra H2 3D imaging System.

**Figure 2:**
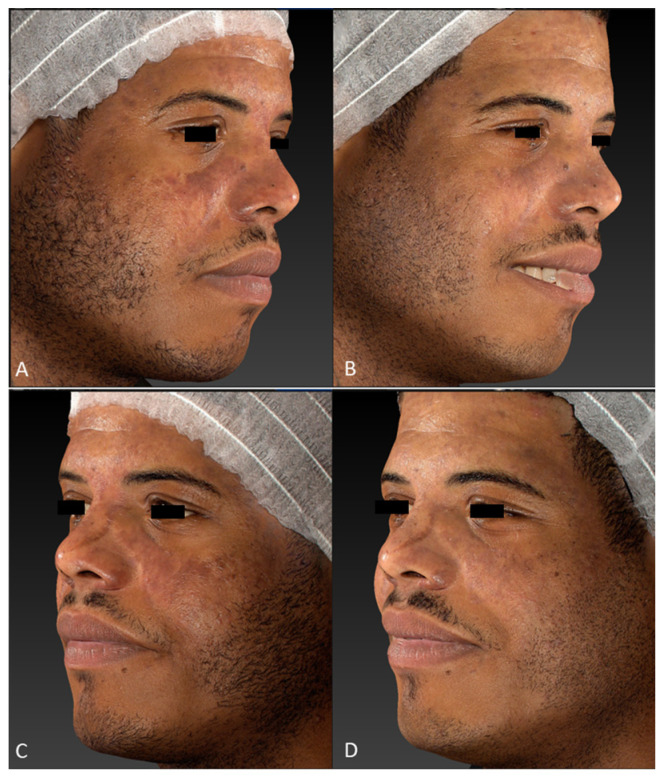
Facial hypermelanoses in a male patient before treatment (right lateral view, (**A**); left lateral view, (**C**)) and three months after the last laser treatment session (right lateral view, (**B**); left lateral view, (**D**)). Images acquired with a Vectra H2 3D imaging System.

**Figure 3:**
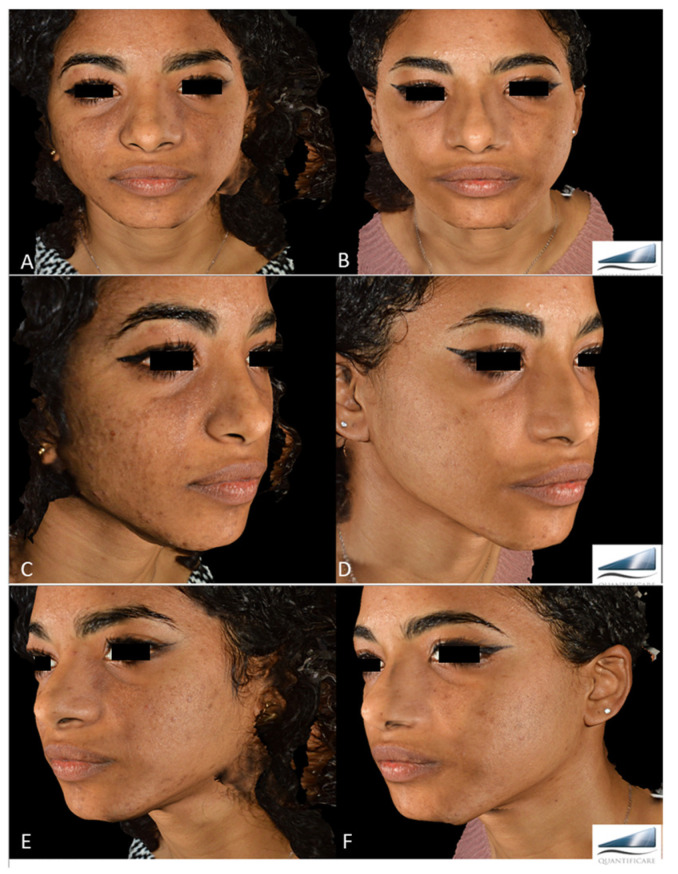
Diffuse Hypermelanoses on a female patient’s face area before treatment (frontal view, **A**; right lateral view, **C**; left lateral view, **E**) and three months after the last laser treatment session (frontal view, **B**; right lateral view, **D**; left lateral view, **F**). Images acquired with a Quantificare 3D imaging System.

**Figure 4:**
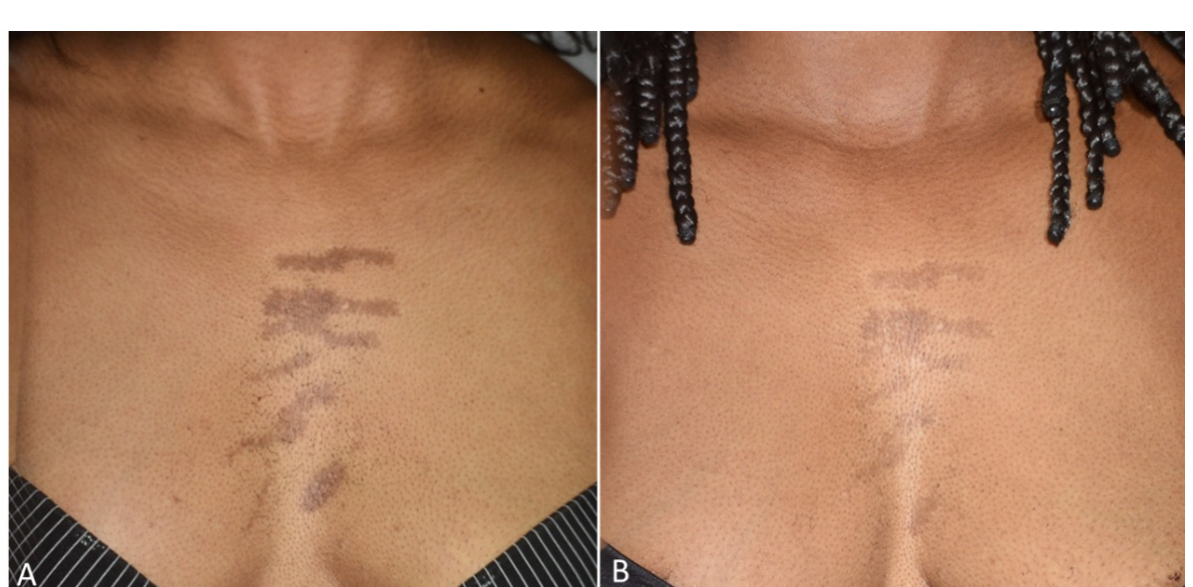
Images of linear hypermelanoses on a female patient’s breast before (**A**) and three months after the last laser treatment session (**B**). A visible clearance of pigmented lesions was observed.

**Figure 5:**
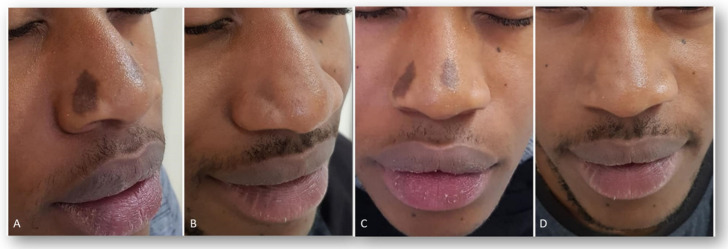
Digital images of two extended hypermelanoses in the nose area of a young male patient, before (lateral view, **A**; frontal view, **C**) and three months after the last laser treatment session (lateral view, **B**; frontal view, **D**). A marked clearance of pigmented lesions was observed.

All patients observed global improvements in their pigmented lesions: 53% of patients achieved excellent clearance, 30% of patients achieved good to moderate clearance, 10% of patients achieved slight clearance, and 7% of patients did not respond to the therapy. No serious adverse events occurred. The prolonged purpura that only two patients with cutaneous hyperpigmentations reported as a side effect spontaneously resolved in two weeks. Treatment was well tolerated by all patients. Following laser treatment for epidermal pigmented lesions, most patients experienced transient oedema and erythema in the perilesional area, which resolved in 1–2 days and was occasionally accompanied by itching. Treatment-related lesions also became darker and were covered in a flake or crusty formation, which exfoliated and eventually transformed into a transient hypopigmentation that resolved completely (no further visible effect) in 30 days. Clinical images (Figure 1, Figure 2, Figure 3, Figure 4 and Figure 5) present the outcomes achieved at 3 months follow-up after the last laser treatment session.

## 4. Discussion

Nowadays, we are increasingly concerned with the appearance of our skin. As such, changes in our pigmentation, such as freckles or pigmentation spots, can be very irritating and have an impact on our well-being. This is the reason why more and more people are choosing to receive treatment for hyperpigmentation. As a result, cosmetic medicine is seeing a rise in the popularity of pigmented lesion removal for conditions like sunspots, freckles, acne blemishes, and other hyperpigmentation [38]. Several treatment options, as mentioned in the Introduction section, are available. Among these, hydroquinone and other bleaching chemicals containing phenolic compounds are frequently employed. Hydroquinone cream can be combined with tranexamic acid, and this combination of therapies has proven to be a significant curative treatment option on patients with complex facial pigmentation [39]. However, adverse outcomes are associated with the use of these compounds. 

The market for laser devices used for this purpose is expanding quickly due to demand. The procedure of eliminating pigmented lesions using laser treatment entails directing a focused laser beam into the skin to eliminate the overabundance of melanin. The primary goal of the procedure is to eliminate or equally distribute the pigment, improving the skin’s texture and appearance [40,41].

Among these conditions, facial hypermelanosis represents a clinical symptom of a wide range of illnesses [42]. 

Since QS lasers work based on selective photo-thermolysis and create a photoacoustic effect, they may manage hypermelanosis. This happens as the lasers produce shock waves that cause targets, like melanin and ink particles, to explode. These targets are then progressively eliminated from the site through blood circulation [20]. 

Melanosome rupture in melanocytes, dermal melanophage destruction, and dermal and epidermal melanosome rupture are among the consequent outcomes [43,44]. Compared to a QSRL 694 nm, a 1064 nm QSNY laser has the advantages of deeper penetration of the photons, due to reduced scattering, and its relative safety in dark skin patients, due to decreased melanin absorption [45]. Several studies have reported the efficacy of QSNY in the treatment of PIH. The superficial distribution of melanin leads all epidermal melanoses to respond favorably to wavelengths of 532 nm [46,47]. Ultrashort 532 nm QS laser systems restrict “collateral thermal injury” to uninvolved peripheral tissue by selectively destroying melanosomes within keratinocytes and melanocytes, therefore enhancing the concept of selective photothermolysis. 

In the treatment of benign hyperpigmentation, there may be treatments that target only melanin, as previously discussed, as well as treatments that target the vascular component, as demonstrated by Temiz et colleagues [48]. This study demonstrated the effectiveness of a 577 nm pro-yellow laser in treating the hyperpigmentation of lesions of Becker’s nevus by acting on the vascular pathogenesis.

Side effects are possible with any aesthetic therapy. The Q-switched lasers provide high safety and only a few adverse effects [49]. The most common complications include hyperpigmentation or hypopigmentation [50], thermal damage [51], and systemic allergic or localized granulomatous tissue reactions [52]. Within weeks of treatment, hypopigmentation appears as little white macules that match the size and form of the laser spot [53]. The probability of hypopigmentation appears to be associated with fluence and the number of treatment sessions [54]. These negative effects, however, can be avoided by employing the appropriate fluence adjusted to the specific patient. Indeed, post-inflammatory hyperpigmentation (PIH) is one of the most frequent side effects of QS laser treatment in individuals with dark skin, even when ideal parameters are followed. Although the precise cause of photoinduced hyperpigmentation is uncertain, direct melanin activation after laser impact is involved. In more recent times, there has been conjecture that QS laser irradiation upregulates melanogenic stimulating factors, including fibroblast growth factors, hepatocyte growth factors, and stem cell factors, which in turn stimulates fibroblasts and increases pigmentation. QS laser therapy is still the most effective treatment for pigmented lesions in people with dark skin because of the mild and temporary nature of PIH [55]. Furthermore, the absorption spectra of most cutaneous laser wavelengths and melanin overlap substantially, and since black skin has higher pigmentation than Caucasian skin, laser therapy for dermal lesions may be hampered by epidermal melanin [56]. Between 532 and 1064 nm—values that are somewhat involved in most commercial lasers on the market today—melanin absorbs light and heat quite strongly. Moreover, keratinocytes with dark skin break down melanosomes more slowly than those with white skin.

Q-switched (QS) laser pulses have been shown to selectively eliminate melanosomes from the skin due to their minimal thermal impact on the adjacent tissues [15]. Nd:YAG and QS ruby lasers have shown promise in treating nevi of Ota and Hori macules, two pigmented lesions that disproportionately affect ethnic groups with darker skin phototypes [57,58,59,60,61,62,63,64]. Fluences should be kept to a minimum in darker skin types to reduce the risk of post-inflammatory hyperpigmentation [65,66,67,68], and a test patch in the covered area may help determine the optimal fluence. Indeed, in our practice, laser treatments are started with a low fluency and gradually increase. In addition, to determine the correct values of the treatment parameters, a skin test is performed before starting the treatment. 

Furthermore, laser therapy should be applied when it is certain that the lesion is benign [15]. Dermoscopy is typically required to examine findings, since it can distinguish potentially worrying lesions. It is imperative to confirm that a pigmented lesion is benign before beginning any physical therapy. When polarized and non-polarized modes are combined, dermatoscopes can display all the characteristics required to differentiate benign from malignant tumors. to avoid a recurrence of the lesions and a delay in diagnosis in the event of malignant lesions, it remains preferable to surgically remove the lesion and avoid treating it with laser therapy when there is doubt about its nature. For instance, when treating a pigmented lesion that presents a thickening around the follicles or rhomboidal pigmented structures, non-homogeneous thickenings, or a blue-grey color or globules under dermatoscopy, instead of using laser therapy, an incisional/excisional biopsy should be carried out.

Only a few published studies regarding the effectiveness of Q-Switched Nd:YAG lasers in the removal of various forms of benign hypermelanosis have been conducted on Asian populations, as reported by Cannarozzo et al. [69]. In this study, the clinical assessment stated a mean score of 2.70 ± 0.78. The mean score was greater for epidermal hyperpigmentation and lower for dermal melanosis, indicating a lesser level of efficacy in the management of the latter kind of lesions. Similar to our study, a quartile scale to assess the lightening of the pigmented lesions was used in these studies.

In addition, the two studies of Vachiramon et al. and Nisticò et al. [22,29], cited in the Introduction section, validate previous findings found in medical literature, emphasizing Q-switched laser treatments as the gold standard for hyperpigmentation.

When comparing the effectiveness and degree of postinflammatory hyperpigmentation from Q-switched Nd:YAG and fractional CO_2_ lasers for treating solar lentigines in Asians, Vachiramon et al.’s research [22] showed that a QS Nd:YAG laser significantly outperformed a fractional CO_2_ laser in terms of lesion lightening. Lesions on the trunk or limbs, as well as superficial melanosis in patients with lighter phototypes, showed a larger response to treatment in the Nisticò et al. research [29]. Conversely, as Silvestri et al. [15] also reported, participants with darker phototypes, facial lesions, and deeper pigmentation disorders responded poorly to the treatment and required a considerable number of sessions to achieve a good result. However, in our research, the selective nanosecond 532 nm wavelength using Fluence of 0.8–1.4 J/cm^2^ and spot size of 3–6 mm was effective in treating darker lesions in dark-skinned individuals, proving to be an innovative study in this field.

Our results clearly showed a global improvement, with 53% of patients who achieved excellent clearance of their pigmented lesions. In this study, no adverse effects among those discussed were detected, and treatment was well tolerated by all patients. As a side effect, only two individuals with dermal hyperpigmentations developed persistent purpura, which spontaneously resolved within two weeks, demonstrating that this technology is safe and effective even for the treatment of dark phototypes, for which few clinical studies are available. 

This innovative QS laser treatment effectively removes the smallest pigment particles in hyperpigmentation. It applies energy using handpieces that have varying profiles to address skin discolorations and pigmented lesions. These new laser devices are the most versatile for efficient treatment with the fewest side effects, due to the various handpiece spot shapes and sizes as well as the various emission modes (pico- and nanoseconds). Furthermore, this laser calculates the amount of melanin in various skin layers using reflectance mapping of many light wavelengths, enabling a uniform measurement of aesthetic results. It delivers energy through handpieces with different profiles to adapt to pigmented lesions and skin discolorations. The different shapes and sizes of the handpiece spots combined with the different emission modes (pico- and nanoseconds) make these new laser devices the most flexible laser systems for effective treatment with minimal contraindications. Additionally, this approach uses reflectance mapping of several various light wavelengths to calculate the quantity of melanin in different skin layers, allowing for a standard measurement of aesthetic outcomes. Our study confirms the results obtained with other double Q-switched lasers, showing exceptional results in the management of hyperpigmentation even in lesions of darker phototypes, which responded excellently to this laser therapy. Furthermore, this technology can be combined with different therapeutic options [70]. Additional prospective and comparative research with a larger sample size of patients could potentially corroborate our findings. 

### Study Limitation

Our study’s limitations include the small number of enrolled patients observed in a limited period of three months, the lack of acquisition of multispectral images of treated areas using the Antera 3D camera, and the lack of histopathogical examination (despite providing a histological test in patients expecting cosmetic outcomes could present complications). 

## 5. Conclusions

Based on this study’s results, Q-switched 1064/532 nm lasers proved to be a key tool for treating benign hypermelanosis in all skin types, including dark-skinned persons. 

## Figures and Tables

**Table 1:** Summary of patient demographics, clinical characteristics of lesions, and treatment protocols.

Number of Patients	% of Female	% of Male	Age Range (Years Old)	Fitzpatrick Skin Types	Lesion Type	Treated Area	Number of Laser Sessions
30	80	20	18–61	IV–V–VI	superficial benign hypermelanoses	Face area and décolleté	1–2

## Data Availability

Data that support the study findings are available on request from the corresponding author (I.F.).
